# Optimizing the Amino Acid Sequence Enhances the Productivity and Bioefficacy of the RBP-Albumin Fusion Protein

**DOI:** 10.3390/bioengineering11060617

**Published:** 2024-06-17

**Authors:** Ji Hoon Park, Sohyun Kwon, So-Young Choi, Bongcheol Kim, Junseo Oh

**Affiliations:** 1 New Drug Development Center, Osong Medical Innovation Foundation, Osong 28160, Republic of Korea; jhpark@kbiohealth.kr (J.H.P.); soyoung@kbiohealth.kr (S.-Y.C.); 2 Department of Biomedical Sciences, College of Medicine, Korea University, Seoul 02841, Republic of Korea; kwonso89@hanmail.net; 3 Senelix Co. Ltd., 25, Beobwon-ro 11-gil, Songpa-gu, Seoul 05836, Republic of Korea; bkim@senelix.com

**Keywords:** fusion protein, fibrosis, linker, protein sequence optimization

## Abstract

The significant growth of the global protein drug market, including fusion proteins, emphasizes the crucial role of optimizing amino acid sequences to enhance the productivity and bioefficacy. Among these fusion proteins, RBP-IIIA-IB, comprising retinol-binding protein in conjunction with the albumin domains, IIIA and IB, has displayed efficacy in alleviating liver fibrosis by inhibiting the activation of hepatic stellate cells (HSCs). This study aimed to address the issue of the low productivity in RBP-IIIA-IB. To induce structural changes, the linking sequence, EVDD, between domain IIIA and IB in RBP-IIIA-IB was modified to DGPG, AAAA, and GGPA. Among these, RBP-IIIA-AAAA-IB demonstrated an increase in yield (>4-fold) and a heightened inhibition of HSC activation. Furthermore, we identified amino acid residues that could form disulfide bonds when substituted with cysteine. Through the mutation of N453S-V480S in RBP-IIIA-AAAA-IB, the productivity further increased by over 9-fold, accompanied by an increase in anti-fibrotic activity. Overall, there was a more than 30-fold increase in the fusion protein’s yield. These findings demonstrate the effectiveness of modifying linker sequences and introducing extra disulfide bonds to improve both the production yield and biological efficacy of fusion proteins.

## 1. Introduction

Tissue fibrosis refers to a pathological condition characterized by the excessive accumulation of extracellular matrix (ECM) components in the organs and tissues, and it can affect various organs, including the liver, pancreas, and kidney [1]. It is widely agreed that myofibroblasts, the primary producers of the ECM proteins observed in fibrotic tissue, predominantly originate from the stellate cells specific to each tissue [2]. For instance, hepatic stellate cells (HSCs) reside within the perisinusoidal space of the liver, constituting approximately 5–8% of all liver cells [3]. These cells, typically quiescent in a healthy liver, exhibit non-proliferative properties and act as reservoirs for ~80% of the body’s vitamin A (retinol) stored as retinyl esters within lipid droplets. Upon the exposure to fibrogenic triggers, quiescent HSCs undergo phenotypic and functional changes, known as “activation,” transitioning into a myofibroblast-like phenotype [4]. During this process, distinct features emerge, including the disappearance of cytoplasmic lipid droplets containing vitamin A, increased cellular proliferation, positive staining for alpha-smooth muscle actin (α-SMA), and an augmented production of ECM proteins. Thus, stellate cell activation plays a crucial role in tissue fibrogenesis and is considered as a promising target for anti-fibrotic therapies [5].

In our previous research, it was observed that albumin expression was present in quiescent stellate cells but absent in the activated ones, albeit at a relatively lower level compared to liver cells [6]. Albumin (~66 kDa), the most prevalent protein in plasma, is composed of three structurally similar domains (I, II, and III), each consisting of two smaller subdomains, A and B [7]. Interestingly, when albumin was forcibly expressed in activated stellate cells, it prompted a significant reversal in the phenotype from myofibroblasts to an early activated state [6]. This led to the reappearance of cytoplasmic lipid droplets and a noticeable decrease in α-SMA and collagen type I expression. These findings led to the development of a novel recombinant fusion protein, combining albumin and retinol-binding protein (RBP), as an effective anti-fibrotic agent [8]. The selection of RBP for targeted delivery to stellate cells stemmed from its pivotal role in facilitating the cellular uptake of retinol within HSCs via the interaction with the membrane receptor called STRA6 [9]. The subsequent mechanistic investigations revealed the importance of intracellular retinoic acid sequestration for the fusion protein’s action [10], prompting the development of an improved fusion protein comprising RBP and albumin domains IIIA and IB (referred to as RBP-IIIA-IB), as these albumin domains reportedly possess high-affinity binding sites for retinoids [7,11]. Studies have shown that RBP-IIIA-IB inhibits stellate cell activation and reduces liver, renal, and pulmonary fibrosis [12,13,14].

Recombinant DNA technology leads the way in generating fusion proteins, introducing a novel category of biomolecules with diverse functionalities. By genetically merging two or more protein domains, these fusion proteins can acquire diverse functions from each component. The successful development of recombinant fusion proteins relies heavily on the careful design of linkers to connect the protein domains [15,16]. In the absence of appropriate linkers, directly fusing functional domains can result in numerous negative outcomes, such as fusion protein misfolding, decreased protein yield, or impaired bioactivity. In this study, we examined the effects of altering the linker sequence and introducing an extra disulfide bond. Our analysis revealed the enhancements in both the production yield and the anti-fibrotic activity of fusion proteins as a result of these modifications. 

## 2. Materials and Methods

### 2.1. Materials 

Male BALB/c mice were purchased from Orient Bio, Inc. (Sungnam, Republic of Korea), and were maintained in environments controlled for temperature, humidity, and lighting. All the animal experimental procedures were approved by our institutional review board (Korea-2021-0119) and were conducted in compliance with the NIH Guide for the Care and Use of Laboratory Animals. ExpiCHO and Expi293 cells were acquired from Thermo Fisher Scientific (Waltham, WA, USA). The synthesis of the fusion protein, RBP-IIIA-IB (depicted in Figure S1), was conducted using a previously established method [8]. In brief, CHO or Expi293 cells underwent transient transfection with plasmids encoding the fusion protein, and the His-tagged fusion protein secreted was purified using Ni Sepharose followed by size-exclusion chromatography.

### 2.2. Isolation of Mouse Hepatic Stellate Cells (HSCs)

HSCs were isolated from male BALB/c mice (14 weeks old) as described previously [17]. In brief, the livers were perfused in situ with phosphate-buffered saline (PBS) and then with Gey’s balanced salt solution (GBSS) supplemented with collagenase (0.5 mg/mL; Sigma-Aldrich, St. Louis, MO, USA) and pronase (1 mg/mL; Sigma-Aldrich). The perfused livers were dissected, and the attached gall bladders and connective tissues were removed. The liver cell suspensions were further digested in GBSS supplemented with collagenase (0.25 mg/mL), pronase (0.5 mg/mL), and DNase (0.07 mg/mL; MP Biomedicals, Santa Ana, CA, USA), for 12 min in a 37 °C water bath. The cells were then washed and centrifuged in a 13.4% Nycodenz gradient at 1400× *g* for 20 min without a break. The interface containing the enriched HSCs was collected and washed with GBSS. Then, the isolated HSCs were cultured in Dulbecco’s modified Eagle’s medium supplemented with 10% fetal bovine serum. The purity of the HSCs was assessed via microscopic observation. The HSCs were passaged before reaching 70% confluence in the primary culture and used as activated HSCs. The activation status of the HSCs was assessed on the basis of their increased expression of α-SMA and collagen type I as well as through their morphologic changes.

### 2.3. Transfection 

ExpiCHO and Expi293 cells were transiently transfected with plasmids using the ExpiFectamine 293 transfection reagent (Thermo Fisher Scientific) according to the manufacturer’s instructions. HSCs were transiently transfected with plasmids using Lipofectamine 2000 (Invitrogen, Carlsbad, CA, USA).

### 2.4. SDS-PAGE and Western Blot Analysis 

Cell lysates were prepared for analyses by electrophoresis and immunoblotting as described previously [12]. The primary antibody against His-tag (Abcam #ab1187, Cambridge, MA, USA) was used.

### 2.5. Quantitative Real-Time PCR 

The total RNA was prepared using TRIzol (Ambion, Austin, TX, USA), and was used to synthesize the cDNA. Real-time PCR was performed on an ABI QuantStudio 3 Real-Time PCR system. To control for the variations in the reaction, the PCR products were normalized against the mRNA levels of glyceraldehyde 3-phosphate dehydrogenase (GAPDH). The primers used were 5′-TATCTGGGAAGGGCAGCAAA-3′ (forward primer) and 5′-CCAGGGAAGAAGAGGAAGCA-3′ (reverse primer) for α-SMA; 5′-GGAGAGTACTGGATCGAC-3′ (forward) and 5′-CTGACCTGTCTCCATGTT-3′ (reverse) for collagen type I; and 5′-GGTGGTCTCCTCTGACTTCAACA-3′ (forward) and 5′-GTTGCTGTAGCCAAATTCGTTGT-3′ (reverse) were used for GAPDH. 

### 2.6. Statistical Analysis 

The results are expressed as the mean ± standard deviation (SD). The paired *t*-test was performed. A *p*-value of less than 0.05 was considered statistically significant.

## 3. Results

### 3.1. The Productivity of the Fusion Protein, RBP-IIIA-IB, Was Low

**Figure 1:**
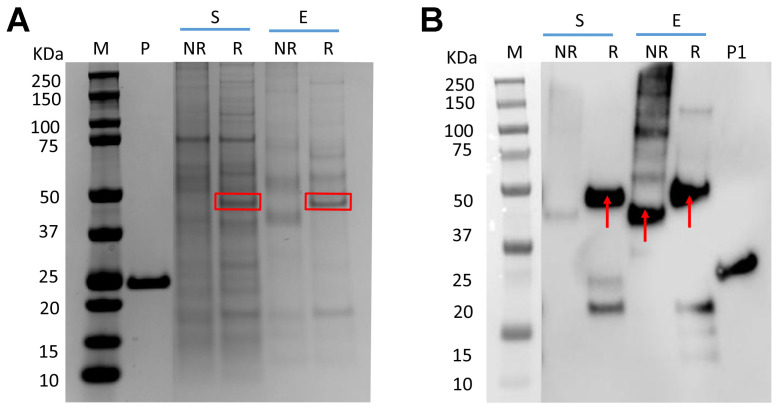
Low productivity of RBP-IIIA-IB in ExpiCHO cells. Following transient transfection of ExpiCHO cells with the plasmid encoding RBP-IIIA-IB, both the culture supernatant and the Ni Sepharose eluate were analyzed via SDS-PAGE (**A**) and Western blotting (**B**). RBP-IIIA-IB is indicated by rectangular box or arrow. S: supernatant, E: Ni-Sepharose eluate, R: reducing condition, NR-non-reducing condition, M: molecular weight marker, P: positive control for protein mass (P: 2 μg, P1: 500 ng).

The fusion protein, RBP-IIIA-IB, carries a signal peptide at its N-terminal for secretion and is fused to a His-tag at its C-terminal for purification, as depicted in Table 1 and Figure S1. After the transient transfection of the plasmid encoding this fusion protein into ExpiCHO cells, the resulting secreted fusion protein (~44 kDa) was purified using Ni Sepharose. SDS-PAGE analysis indicated that both the fusion protein yield and the purity of the nickel eluate were low, suggesting that producing RBP-IIIA-IB in its current form is not advisable (Figure 1A). Furthermore, Western blot analysis using an anti-His tag antibody revealed that a substantial amount of the fusion protein existed in an aggregated state and that the low yield was not primarily due to protein degradation (Figure 1B).

### 3.2. Altering the Linking Sequence between IIIA and IB Enhanced the Productivity and Bioactivity of RBP-IIIA-IB

**Figure 2:**
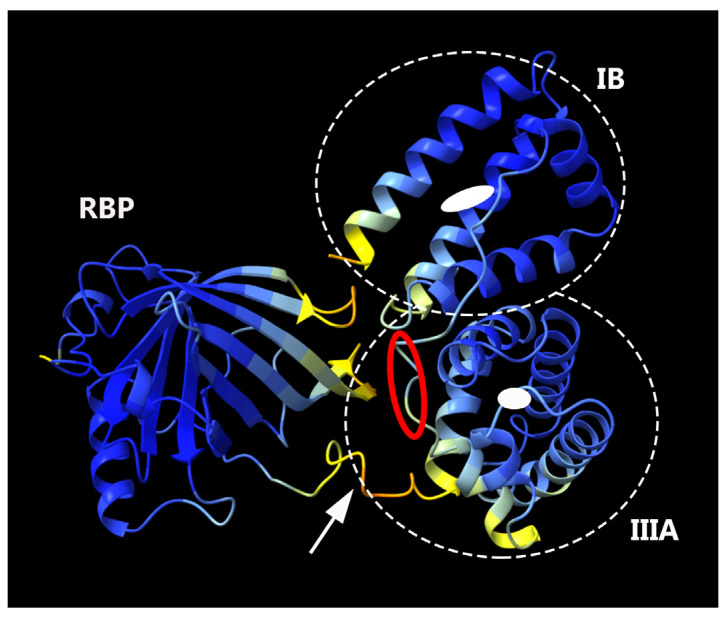
The predicted structure of RBP-IIIA-IB as determined by AlphaFold2. Within this structure, the domains RBP and albumin IIIA and IB are discernible, with the connecting region between RBP and IIIA highlighted by a white arrow. The binding pockets for retinoic acid in domains IIIA and IB are illustrated by white closed circles, and the region of the modified linker sequence is indicated with a red circle.

**Figure 3:**
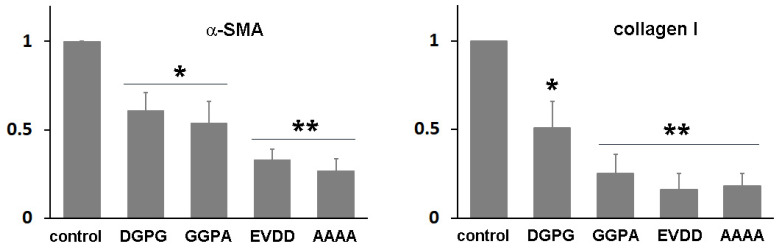
The expression of RBP-IIIA-linker-IB inactivated hepatic stellate cells (HSCs). HSCs after passage 1 were transiently transfected with a plasmid encoding a fusion protein containing different linker sequences, and the levels of alpha-smooth muscle actin (α-SMA) and collagen type I were evaluated using real-time PCR. The data represent the means ± standard deviation of three independent experiments. * *p* < 0.05, ** *p* < 0.01, paired *t*-test (compared with control cells).

We employed AlphaFold2 to predict the structure of the fusion protein. The predicted structure displays a relatively compact conformation, achieving a confidence score exceeding 90% (Figure 2). Notably, the connecting region between RBP and IIIA appears flexible, as highlighted by a white arrow. We hypothesized that this compact, globular form might lead to physical hindrance to the activity of the individual protein components and could also contribute to the protein aggregation. To investigate this possibility, the linking sequence, EVDD, between IIIA and IB (highlighted in red in Table 1, and marked with a red circle in Figure 2) was modified. We chose linkers that were neither flexible nor rigid, ensuring that they could maintain the proper spacing between domains IIIA and IB while preserving the native conformation of each domain, as predicted by AlphaFold2. Examples of such substitutions include AAAA, DGPG, and GGPA. The hepatic stellate cells at passage 1 (HSCs-P1; activated HSCs) were transiently transfected with a plasmid encoding a fusion protein that incorporates various linking sequences. Subsequently, real-time PCR was employed to assess the mRNA expression levels of α-SMA and collagen type I (Col1a1), both well-established markers for activated HSCs [18]. The expression of the fusion protein led to a decrease in the mRNA levels of these markers (Figure 3). Notably, among them, the fusion protein incorporating the AAAA linker (referred to as RBP-IIIA-AAAA-IB) exhibited the most significant efficacy in inactivating HSCs. This reduction in α-SMA expression was also confirmed via Western blotting (Figure S2).

**Figure 4:**
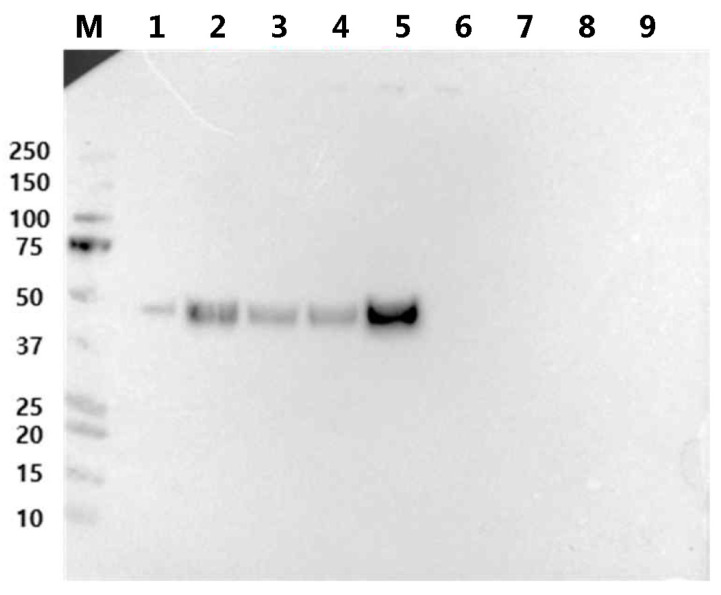
The protein expression levels of RBP-IIIA-linker-IB and IIIA-linker-IB-RBP in Expi293 cells. Expi293 cells were transiently transfected with a plasmid encoding the fusion protein containing various linking sequences, and an equal amount of culture supernatant was subjected to Western blot analysis using an anti-His tag antibody. Lane 1: positive control for protein mass, 2: RBP-IIIA-DGPG-IB, 3: RBP-IIIA-GGPA-IB, 4: RBP-IIIA-EVDD-IB, 5: RBP-IIIA-AAAA-IB, 6: IIIA-DGPG-IB-RBP, 7: IIIA-GGPA-IB-RBP, 8: IIIA-EVDD-IB-RBP, 9: IIIA-AAAA-IB-RBP, M: molecular weight marker.

**Figure 5:**
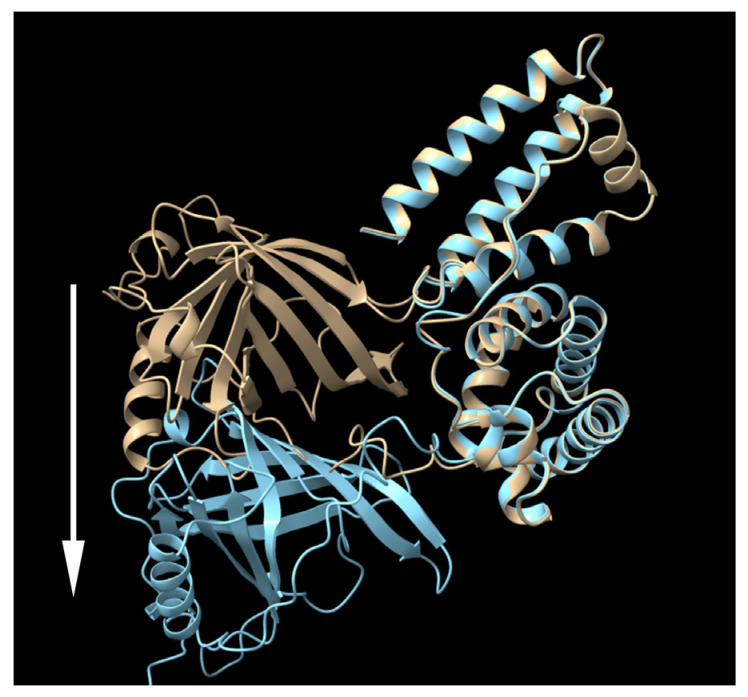
The predicted structure of RBP-IIIA-EVDD-IB (yellow) and RBP-IIIA-AAAA-IB (cyan) as determined by AlphaFold2. RBP. It is notable that the RBP component in RBP-IIIA-AAAA-IB shifts away from the RBP of RBP-IIIA-EVDD-IB, indicated by the white arrow.

Following this, Expi293 cells were transiently transfected with a plasmid encoding a fusion protein, and the culture supernatant was analyzed via Western blotting. Among the tested fusion proteins, RBP-IIIA-AAAA-IB was the most abundantly produced, showing over a four-fold increase compared to the original fusion protein, RBP-IIIA-EVDD-IB (Figure 4). In this study, both ExpiCHO and Expi293 cell lines were used to confirm that there are no significant disparities in protein productivity between them. However, a direct comparison of the protein productivity between the two cell lines was not conducted. Additionally, we investigated whether altering the orientation of the protein components by positioning RBP at the C-terminal end (Figure S1) could augment the expression of fusion proteins. However, we observed restricted expression levels (Figure 4). In line with the findings from both the protein yield assessment and real-time PCR, only RBP-IIIA-AAAA-IB exhibited an elongated conformation as predicted by AlphaFold2 (Figure 5). Consequently, RBP-IIIA-AAAA-IB was selected for further investigation.

### 3.3. The Productivity of RBP-IIIA-AAAA-IB Was Further Enhanced by the Insertion of an Additional Disulfide Bond

**Figure 6:**
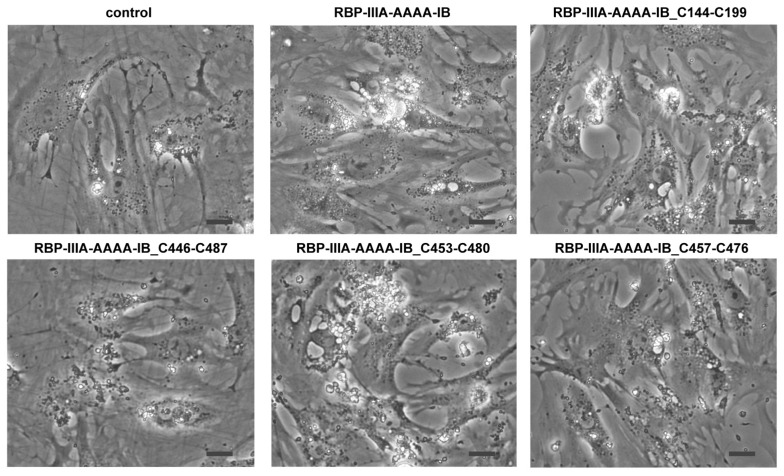
The expression of RBP-IIIA-AAAA-IB with an additional disulfide bond induced phenotypic changes in hepatic stellate cells (HSCs). HSCs after passage 1 were transiently transfected with a plasmid encoding the fusion protein featuring cysteine substitutions at T446-L487, N453-V480, V457-Y476, or V144-A199, and their morphology was observed using a light microscope. Scale bar, 20 μm.

**Figure 7:**
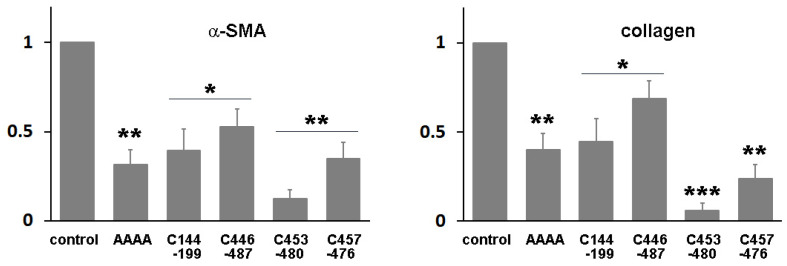
The expression of RBP-IIIA-AAAA-IB with an additional disulfide bond inactivated hepatic stellate cells (HSCs). HSCs after passage 1 were transiently transfected with a plasmid encoding the fusion protein featuring cysteine substitutions at T446-L487, N453-V480, V457-Y476, or V144-A199, and the levels of alpha-smooth muscle actin (α-SMA) and collagen type I expression were assessed using real-time PCR. * *p* < 0.05, ** *p* < 0.01, *** *p* < 0.001, paired *t*-test (*n* = 3) (compared with control cells).

Domain IIIA of albumin contains four native disulfide bonds, while domain IB has two. To investigate the potential enhancement of the structural stability and subsequent yield increase by introducing an extra disulfide bond [19], we identified amino acid residues that may form disulfide bonds when substituted with cysteine. Cysteine substitutions were conducted at positions V144-A199 (the number denotes the position of the amino acid residue within albumin) (Table 1), T446-L487, N453-V480, or V457-Y476 (Figure S3). Subsequently, we evaluated the biological activity of the resulting mutant proteins in HSCs to examine their structural impact. When HSCs-P1 were transiently transfected with a plasmid encoding each variant, they exhibited significant phenotypic changes, including the reappearance of lipid droplets, cell shrinkage, and reduced stress fibers (Figure 6). Among these variants, the fusion protein RBP-IIIA-AAAA-IB with N453C-V480C (referred to as RBP-IIIA-AAAA-IB_C453-480) and RBP-IIIA-AAAA-IB_C457-476 exhibited pronounced alterations in their morphology. Furthermore, real-time PCR analysis revealed decreased mRNA expression levels of α-SMA and collagen type I with the expression of fusion proteins, particularly with RBP-IIIA-AAAA-IB_C453-480 displaying the most significant effect (Figure 7). Western blotting also confirmed the decrease in α-SMA expression (Figure S4). These results suggest that disulfide-stabilized fusion proteins elicit enhanced biological activity.

**Figure 8:**
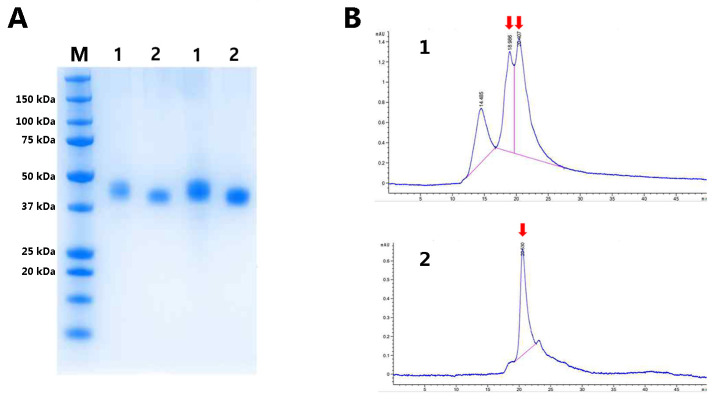
The synthesis of RBP-IIIA-AAAA-IB and RBP-IIIA-AAAA-IB_C453-480 in Expi293 cells. Expi293 cells were transiently transfected with the plasmid encoding either RBP-IIIA-AAAA-IB (1) or RBP-IIIA-AAAA-IB_C453-480 (2), and the resulting fusion proteins were purified through Ni Sepharose and size exclusion chromatography. SDS-PAGE analysis was performed on the purified fusion protein samples (**A**), with the corresponding size exclusion chromatography profile displayed (**B**). M, molecular weight marker.

**Figure 9:**
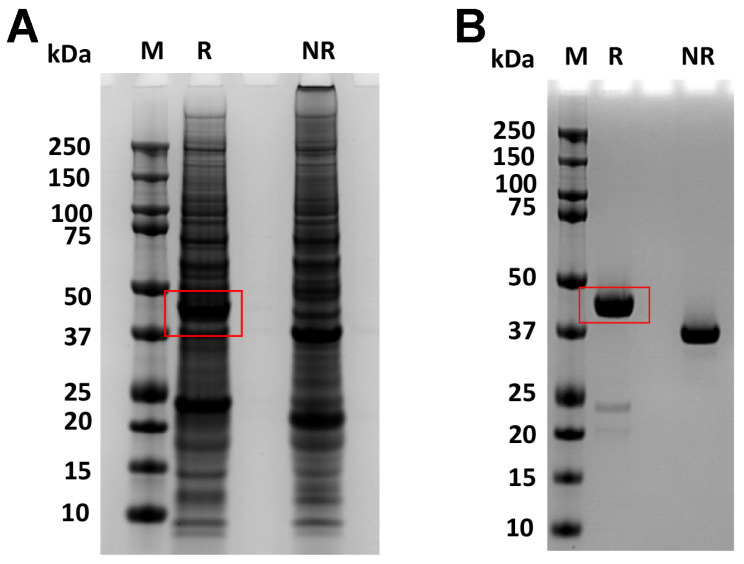
The increased production of RBP-IIIA-AAAA-IB_C453-480 in ExpiCHO cells. After ExpiCHO cells were transiently transfected with the plasmid encoding RBP-IIIA-AAAA-IB_C453-480, both the culture supernatant (**A**) and the purified fusion protein (**B**) were analyzed using SDS-PAGE. M, molecular weight marker; R, reduction; NR, non-reduction.

Subsequently, Expi293 cells were transiently transfected with a plasmid encoding either RBP-IIIA-AAAA-IB or RBP-IIIA-AAAA-IB_C453-480, and the resulting fusion proteins were purified using Ni Sepharose followed by size exclusion chromatography. SDS-PAGE analysis demonstrated a slightly blurred band corresponding to RBP-IIIA-AAAA-IB (Figure 8A), suggesting the possible fragility in its physical properties, a feature further supported by the size exclusion chromatography profile (Figure 8B). The evaluation of the protein yield revealed that the protein yield of RBP-IIIA-AAAA-IB_C453-480 was over nine times higher than that of RBP-IIIA-AAAA-IB. Finally, ExpiCHO cells were transiently transfected with the plasmid encoding RBP-IIIA-AAAA-IB_C453S-480. The resulting fusion proteins were purified and subjected to SDS-PAGE. Notably, there was a significant increase in productivity observed (Figure 9). The evaluation of the protein yield in ExpiCHO cells showed that the protein yield of RBP-IIIA-AAAA-IB_C453-480 exceeded that of RBP-IIIA-EVDD-IB by over 30 times, similar to the outcomes obtained from Expi293 cells. This finding suggests that the introduction of an additional disulfide bond contributes to an elevation in protein yield, possibly through bolstering the protein’s stability.

### 3.4. The Fusion Protein RBP-IIIA-AAAA-IB_C453-480 Showed Improved Anti-Fibrotic Activity

**Figure 10:**
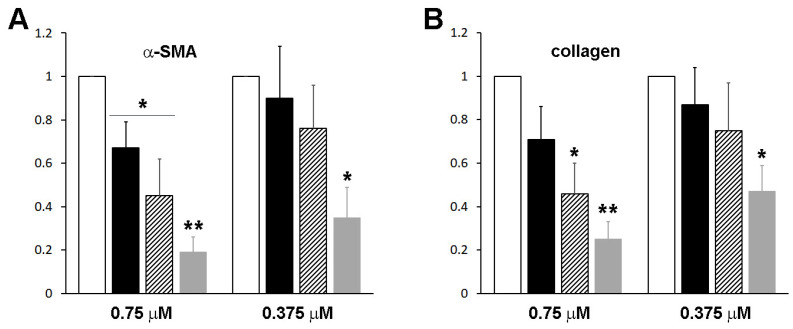
The anti-fibrotic effects of the fusion proteins on hepatic stellate cells (HSCs). HSCs after passage 1 were treated with purified fusion proteins (white), RBP-IIIA-EVDD-IB (black) (0.75 or 0.375 μM), RBP-IIIA-AAAA-IB (hatched), or RBP-IIIA-AAAA-IB_C453-480 (gray), for 16 h, and the levels of alpha-smooth muscle actin (α-SMA) (**A**) and collagen type I (**B**) expression were evaluated using real-time PCR. * *p* < 0.05, ** *p* < 0.01, paired *t*-test (*n* = 3) (compared with control cells).

**Figure 11:**
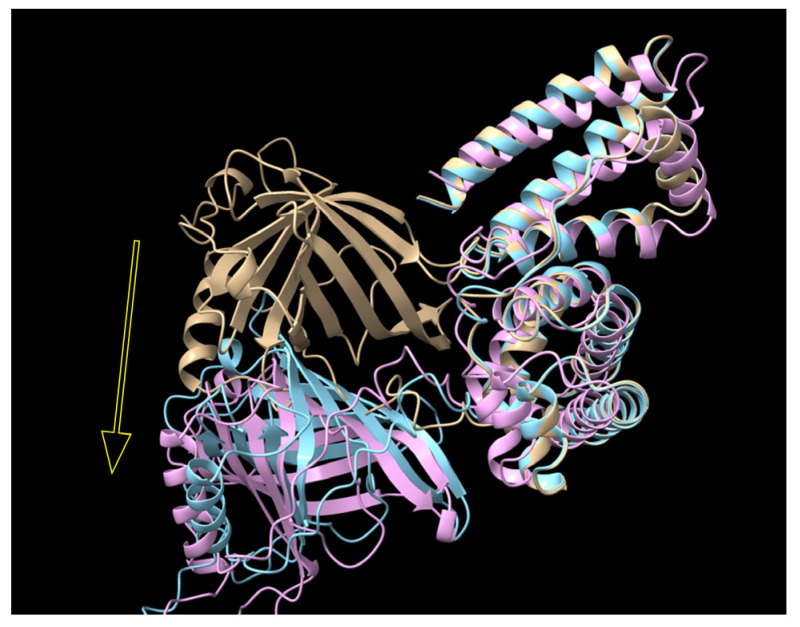
The predicted structure of RBP-IIIA-EVDD-IB (yellow), RBP-IIIA-AAAA-IB (cyan), and RBP-IIIA-AAAA-IB_C453-480 (pink) as determined by AlphaFold2. RBP. It is notable that RBP components in RBP-IIIA-AAAA-IB and RBP-IIIA-AAAA-IB_C453-480 gradually diverge from the RBP of RBP-IIIA-IB, indicated by the yellow arrow.

To enhance the protein productivity, we altered the amino acid sequence of the fusion protein, progressing from RBP-IIIA-EVDD-IB to RBP-IIIA-AAAA-IB and subsequently to RBP-IIIA-AAAA-IB_C453-480. We then evaluated the anti-fibrotic properties of these three fusion proteins on activated HSCs *in vitro*. Following treatment of HSCs-P1 with purified fusion proteins, we measured the expression levels of α-SMA and collagen type I using real-time PCR. RBP-IIIA-AAAA-IB_C453-480 exhibited the most pronounced effects on their mRNA levels, with RBP-IIIA-AAAA-IB ranking second (Figure 10). Western blotting also confirmed the decrease in α-SMA expression (Figure S5). When comparing the predicted structures of these fusion proteins using AlphaFold2, it is noteworthy that the RBP components in RBP-IIIA-AAAA-IB and RBP-IIIA-AAAA-IB_C453-480 gradually shifted away from the RBP of RBP-IIIA-EVDD-IB (Figure 11). The initial compact shape of the protein evolved into an elongated form. These findings suggest that the substitution of the linking sequence, EVDD, between albumin domains IIIA and IB with AAAA, along with the addition of an extra disulfide bond at C453-480, synergistically enhanced both the productivity and anti-fibrotic activity, likely by reshaping and fortifying the protein structure.

## 4. Discussion

Producing fusion proteins is complex due to the diverse physiochemical properties inherent in proteins. The structural disparities between protein components may cause misfolding, instability, and often lead to low expression yields or protein aggregation [20]. Overcoming the challenge of achieving stable and high expression levels of recombinant fusion proteins poses a significant obstacle in their therapeutic development. Linkers, essential elements of recombinant fusion proteins, are gaining more recognition for their pivotal role in fusion protein development [15,21]. Beyond enhancing the stability, linkers can provide various benefits in fusion protein production, such as boosting the biological activity and increasing the expression yields [22,23,24].

After observing a low yield in RBP-IIIA-EVDD-IB and noticing that its structure, as predicted by AlphaFold2, exhibited a relatively compact form, we sought to adjust the linking sequences between the protein components. Given that the connecting region between RBP and IIIA appeared adequately flexible, we decided to modify the linking sequence, EVDD, between albumin domains IIIA and IB. Flexible linkers are frequently considered when the connected components require a degree of movement or interaction. These linkers often contain abundant small or polar amino acids like Gly and Ser, while also incorporating additional amino acids such as Thr and Ala to preserve the flexibility [25]. Flexible linkers have been employed to enhance both yield and biological activity. However, the literature also documents cases where their use led to poor expression yields or compromised biological activity [24,26]. This inefficacy was attributed to the inadequate separation of protein components or insufficient reduction in the interference between them. In such cases, rigid linkers can be considered to maintain a fixed distance between domains and preserve their independent functions. According to George and Heringa [27], many rigid linkers exhibit stiff structures through the adoption of α-helical structures or the incorporation of multiple Pro residues, effectively separating the functional domains more efficiently than flexible linkers. In this study, we examined various flexible and rigid linkers, yet AlphaFold2 analysis deemed them unsuitable for RBP-IIIA-IB. Subsequently, we chose to use mixed linkers and assessed their impact on the production yield and bioefficacy. Among the linkers examined, only the AAAA linker caused changes in the protein structure from compact to elongated, as projected by AlphaFold2. RBP-IIIA-AAAA-IB showed an improved yield and HSC-inactivating activity. Nonetheless, the exact mechanism underlying the alterations induced by the modified linker remains unclear. 

In order to further improve both the stability and biological activity of RBP-IIIA-AAAA-IB, we generated four mutant proteins with cysteine substitutions. An earlier study demonstrated that incorporating the artificial insertion of a disulfide bond may be applied to bolster the structure and promote bioefficacy [28,29]. Among these mutants, RBP-IIIA-AAAA-IB_C453-480 showed a significantly increased yield and anti-fibrotic efficacy, suggesting that the extra disulfide bond between helix 3 and helix 4 within domain IIIA enhanced the protein stability. The positioning of the disulfide bond may yield varying effects on the protein stability. The verification of disulfide bond formation at the engineered site in C454-480 is necessary, possibly utilizing high-resolution mass spectrometry. 

We employed AlphaFold2 to predict the structure of the fusion protein, aiming to comprehend the reasons for its low yield and identify the effective solutions. AlphaFold2 assesses its predictions according to their confidence levels, with the first prediction being the most confident or highest-scoring structure. Although the confidence score reflects the model’s certainty level, it does not assure absolute accuracy [30]. Hence, it is crucial to recognize that the predicted structure should undergo evaluation and validation based on the structural coherence and biological relevance. 

We assessed the yield and biological efficacy following each alteration in the amino acid sequence. When compared to RBP-IIIA-EVDD-IB, RBP-IIIA-AAAA-IB exhibited more than a 4-fold increase in yield in Expi293 cells and enhanced the HSC inactivating activity. Subsequently, RBP-IIIIA-AAAA-IB_C453-480 demonstrated a yield enhancement of over 9-fold compared to RBP-IIIA-AAAA-IB in Expi293 cells. In ExpiCHO cells, RBP-IIIIA-AAAA-IB_C453-480 showed a yield enhancement of over 30-fold compared to RBP-IIIA-EVDD-IB, which aligns with the finding in Expi293 cells.

Therefore, this study illustrates the intricacies associated with the production of fusion proteins. Our research represents an example of the protein rational design, where the fusion protein undergoes modification through linker optimization and the insertion of disulfide bonds, leading to the enhancement not only in yield but also in biological activity.

## 5. Conclusions

Our findings demonstrate that modifying the linking sequence (EVDD→AAAA) between albumin domains IIIA and IB in the fusion protein, RBP-IIIA-IB, leads to an increased production yield and the improved inhibition of HSC activation. Moreover, introducing an extra disulfide bond results in a further boost in yield and bioefficacy. These results highlight the importance of optimizing the amino acid sequences in the advancement of fusion protein drugs.

## 6. Patents

The experimental findings have been submitted for patent (10-2023-0012988) to the South Korea Patent Office.

## Figures and Tables

**Table 1:** The amino acid sequence of RBP-IIIA-IB.

Component	Amino Acid Sequence
RBP (1-193)	mkwvwallllaawaaaERDCRVSSFRVKENFDKARFSGTWY AMAKKDPEGLFLQDNIVAEFSVDETGQMSATAKGRVR LLNNWDVCADMVGTFTDTEDPAKFKMKYWGVASFLQ KGNDDHWIVDTDYDTYAVQYSCRLLNLDGTCADSYSF VFSRDPNGLPPEAQKIVRQRQEELCLARQYRLIVHNGY CDGR
albumin IIIA (404-517)	L VEEPQNLIKQNCELFEQLGE YKFQNALLVRYTKKVPQVST PTLVEVSRNLGKVGSKCCKH PEAKRMPCAEDYLSVVLNQL CVLHEKTPVSDRVTKCCTES LVNRRPCFSALEV
albumin IB (131-218)	DDNPNLPRLVRPEV DVMCTAFHDNEETFLKKYLY EIARRHPYFYAPELLFFAKR YKAAFTECCQAADKAACLLP KLDELRDEGKASSA hhhhhhhh

The signal peptide sequence and His-tag are in lowercase letters. The numbers in parentheses indicate the positions of the amino acid residues within RBP or albumin.

## Data Availability

The data presented in this study are available on request from the corresponding author.

## Supplementary Materials

The following supporting information can be downloaded at: https://www.mdpi.com/article/10.3390/bioengineering11060617/s1, Figure S1: Schematic diagram of the retinol-binding protein (RBP)–albumin domain IIIA-IB fusion proteins. Figure S2. Hepatic stellate cells at passage 1 were transiently transfected with a plasmid encoding a fusion protein with different linking sequences and analyzed via Western blotting using antibodies against α-SMA or α -tubulin. Lane 1: control, 2: DGPG, 3: GGPA, 4: EVDD, 5: AAAA. Figure S3. The predicted structure of RBP-III-AAAA-IB as determined via AlphaFold2. The locations where extra disulfide bonds are inserted are marked in red (C144-199), green (C446-487), yellow (C453-480), and white (C457-476). Figure S4. HSCs after passage 1 were transiently transfected with a plasmid encoding the fusion protein featuring cysteine substitutions at V144-A199, T446-L487, N453-V480, or V457-Y476, and analyzed via Western blotting using antibodies against α-SMA or α-tubulin. Lane 1: control, 2: V144-A199, 3: T446-L487, 4: N453-V480, 5: V457-Y476. Figure S5. HSCs after passage 1 were treated with purified fusion proteins (0.375 μM) and analyzed via Western blotting using antibodies against α-SMA or α -tubulin. Lane 1: control, 2: RBP-IIIA-EVDD-IB, 3: RBP-IIIA-AAAA-IB, 4: RBP-IIIA-AAAA-IB_C453-480.
