# Supplementary material for: Optimizing the Amino Acid Sequence Enhances the Productivity and Bioefficacy of the RBP-Albumin Fusion Protein
Source: Bioengineering (Basel). 2024 Jun 17;11(6):617. doi: 10.3390/bioengineering11060617 (PMC11200973; doi:10.3390/bioengineering11060617)

Supplementary Figure S1. A schematic diagram illustrates the fusion protein composed of retinol-binding protein (RBP) and albumin domains IIIA and IB, alongside full-length albumin and RBP for comparison. Note that the fusion protein is tagged with histidine at the C-terminal end, with amino acids indicated by numbers.

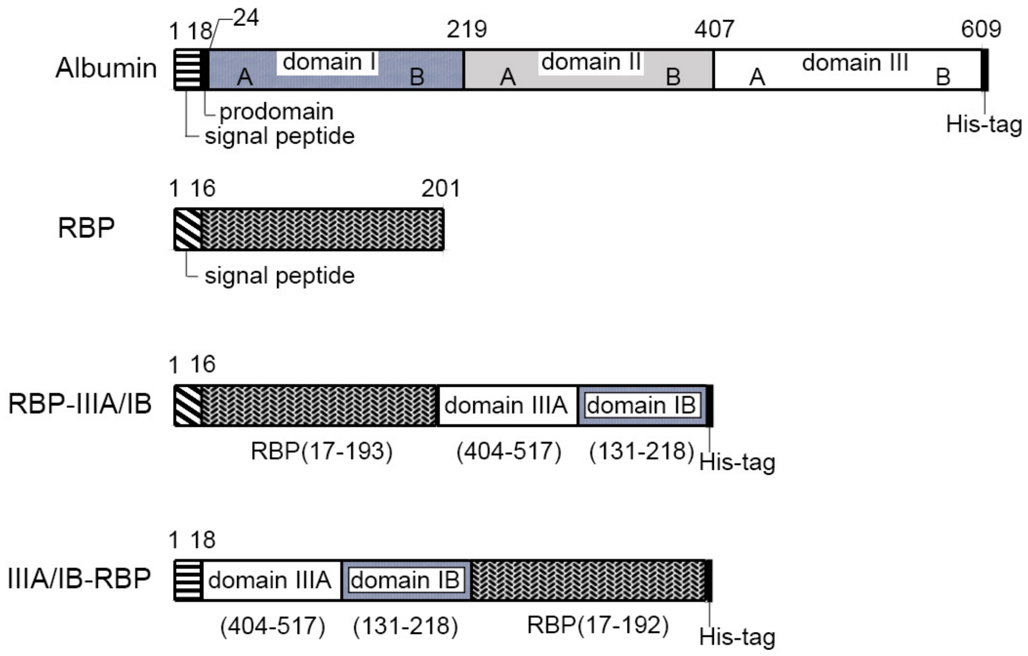

Supplementary Figure S2. Hepatic stellate cells at passage 1 were transiently transfected with a plasmid encoding a fusion protein with different linking sequences and analyzed by Western blotting using antibodies against α-SMA or α-tubulin. Lane 1: control, 2: DGPG, 3: GGPA, 4: EVDD, 5: AAAA.

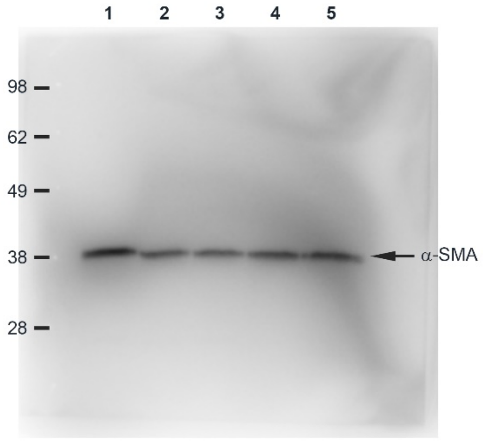

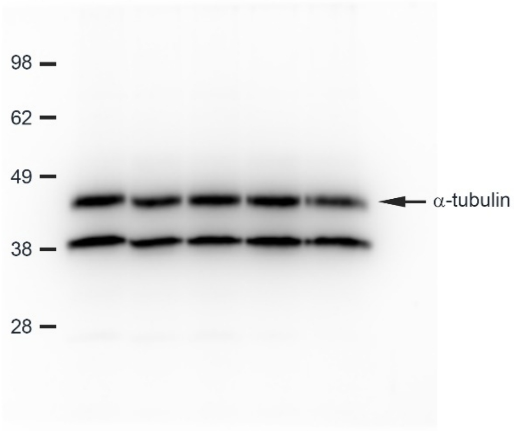

Supplementary Figure S3. The predicted structure of RBP-III-AAAA-IB as determined by AlphaFold2. The locations where extra disulfide bonds are inserted are marked in red (C144-199), green (C446-487), yellow (C453-480), and white (C457-476).

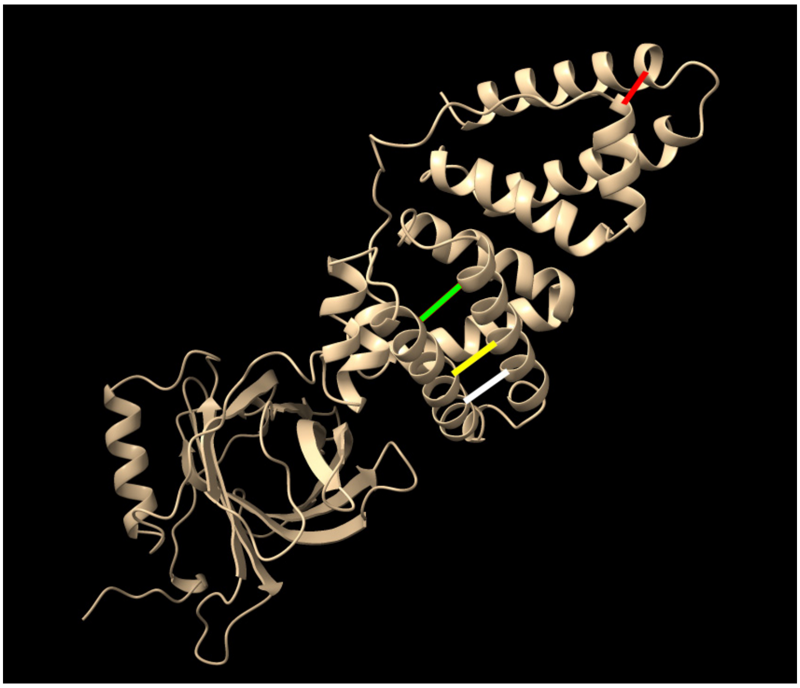

Supplementary Figure S4. HSCs after passage 1 were transiently transfected with a plasmid encoding the fusion protein featuring cysteine substitutions at V144-A199, T446-L487, N453-V480, or V457-Y476, and analyzed by Western blotting using antibodies against α-SMA or α-tubulin. Lane 1: control, 2: V144-A199, 3: T446-L487, 4: N453-V480, 5: V457-Y476.

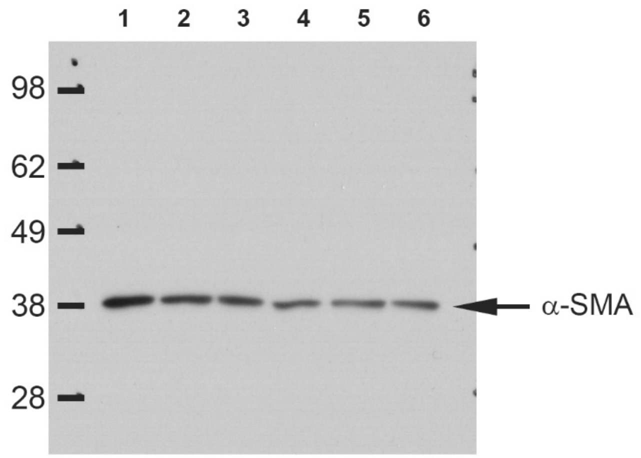

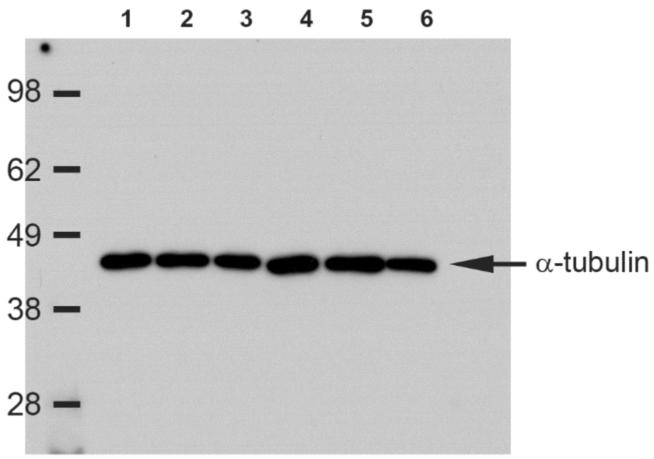

Supplementary Figure S5. HSCs after passage 1 were treated with purified fusion proteins (0.375 μM) and analyzed by Western blotting using antibodies against α-SMA or α-tubulin. Lane 1: control, 2: RBP-IIIA-EVDD-IB, 3: RBP-IIIA-AAAA-IB, 4: RBP-IIIA-AAAA-IB_C453-480.

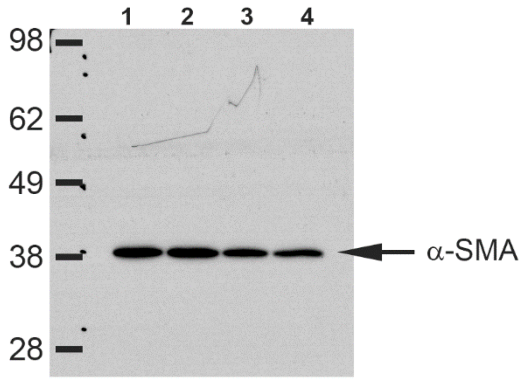

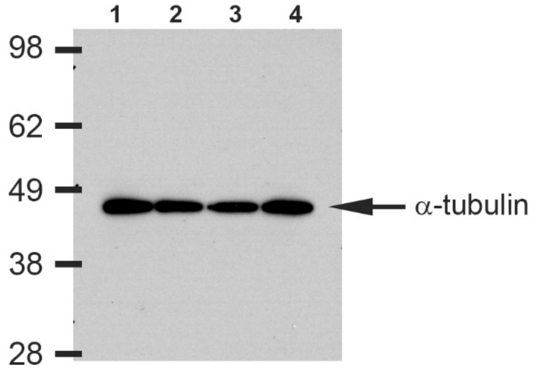

Supplement: Supplementary file 1 [file bioengineering-11-00617-s001.zip › bioengineering-3060981-supplementary.pdf]
